# Mental health among immigrants in Germany – the impact of self-attribution and attribution by others as an immigrant

**DOI:** 10.1186/s12889-019-8060-y

**Published:** 2019-12-18

**Authors:** Yuriy Nesterko, Michael Friedrich, Elmar Brähler, Andreas Hinz, Heide Glaesmer

**Affiliations:** 1 Department of Medical Psychology and Medical Sociology, University of Leipzig, Philipp-Rosenthal-Str. 55, 04103 Leipzig, Germany; 2 Clinic for Psychosomatic Medicine and Psychotherapy, University of Mainz, Mainz, Germany

**Keywords:** Immigration, Self-attribution, PTSD, Anxiety, Depression

## Abstract

**Background:**

In Germany, the term ‘migration background’ has been established to differentiate between immigrants and natives. In the present study post-traumatic stress disorder (PTSD), anxiety, and depression were analysed in immigrant populations in Germany by considering self-attribution as well as attribution by others on one’s own ‘migration background’.

**Methods:**

In a population-based survey (*N* = 2317), socio-demographic characteristics, migration background (official statistics definition vs. self-attribution as well as the anticipated attribution by others), PTSD (PCL-5), and symptoms of anxiety and depression (PHQ-4) were assessed. Logistic regression models were applied to predict mental health outcomes by considering socio-demographic and immigration-related factors.

**Results:**

A total of 10.7% of respondents (*N* = 248) had a ‘migration background’. Immigrants of the 2nd generation compared to 1st generation immigrants are less likely to see themselves as immigrants. Attribution as an immigrant (self and/or by others) was found as significant predictor for PTSD and depression, but not anxiety.

**Conclusions:**

It seems useful to focus on immigration-related factors considering subjective perspectives and not only comparing immigrants and natives using a federal statistics definition. Our findings suggest that research on the association between immigration-related factors such as attribution as an immigrant and mental health outcomes might be a promising approach to better identify subgroups at higher risk of mental distress.

## Introduction

The impact of immigration - as a multidimensional, long-term process - on mental health status of immigrants has been repeatedly demonstrated in empirical studies over the last few decades [1–3]. Accordingly, it has often been noted that the immigration process is associated with hardships, difficulties, and in its consequence with a higher vulnerability to mental health problems [4–6], even though direction, type, and level of severity of the relationship health and migration are very complex. However, there are studies indicating better mental health in different groups of immigrants compared with the native populations [1, 7], whereas other studies report worse mental health outcomes among different groups of immigrants [8–10]. The findings are often contradictory and thus might be attributed to differences in respect to the methodology of each study [3]. In Germany, a country with a high proportion of immigrants, the term ‘person with a migration background’, has been established to differentiate between immigrants and natives. It is a criteria-based definition including the information on nationality, country of birth, and naturalization including both 1st and 2nd generation immigrants.

In international migration research, especially in Anglo-American regions, other concepts are used. The terms ‘ethnicity’ or ‘race’ are particularly common, although it should be noted that the history of immigration in the major host countries such as the U.S., Canada, Australia, Great Britain, France or Germany, as well as the current immigration movements to these countries are not outrightly comparable [11, 12]. In the U.S., for example, White Americans are particularly often compared with African and/or Hispanic Americans, rather than focusing on ethnicity [13]. The terms ‘ethnicity’, ‘foreigners’ and ‘immigrants’, which are frequently used in Europe, including Germany, are often still undifferentiated, i.e. understood and used synonymously, although they differ greatly in content and respectively in the way they are operationalized. While the ethnic affiliation to a group is based on language, culture, and/or religion and thus addresses subjectively reflected experiences [14], ‘foreigners’ and ‘immigrants’ are rather formal terms being defined by objective criteria. This is exactly where important questions arise which have not been addressed in international migration research until now: (1) is there an overlap between the officially used definition and the subjective attribution to one’s own ‘migration background’ or rather immigration status and (2) how do possible interactions in this regard relate to mental health in different immigrant populations?

In this light, a differentiation between objective and subjective perspectives on immigration status definition seems important, especially due to the common use of immigration status as a potential risk factor in numerous research areas. A particularly striking example of this is research on perceived discrimination in the context of well-being and mental health. There is evidence that experiences of discrimination have a negative impact on mental health of those who have been affected or discriminated against [15–19]. If perceived discrimination is linked to the usual assessment of immigration status according to the objective, criteria-based definition, a distortion or underestimation of the effect could occur: individuals who do not perceive themselves as immigrants would not interpret an experience of discrimination to their existing immigration status according to official definition or would therefore not feel discriminated against. In addition, another important dimension in this context, which has hardly been investigated so far, is the question of one’s own social, cultural and/or ethnic identity having an impact on mental health. Whereas there is consensus about negative association between perceived discrimination and psychological well-being as well as physical and mental health [20], contrary findings on the relationship between minority group identification and psychological well-being are available. Crabtree et al. [21] found a negative direct link between group identification and well-being among mentally ill participants. Hence, the authors argued that identification with stigmatized groups may not always have a protective role. In contrast, Greenaway et al. [22] conclude more generally that people who are highly identifying with their social group show better mental and physical health. However, it is also discussed that group identification leads to more pronounced discrimination, thus having a negative impact on psychological well-being [23]. Consequently, it seems important to assess subjective attributions in addition to the criteria-oriented, official definition with respect to one’s own immigration status to investigate the differential impact of subjective perception as well as its potential impact on mental health.

In the present study, the relationship between immigration status (German natives vs. 1st vs. 2nd generation immigrants) and a differentiation of subjective perception of one’s own official ‘migration background’ (assessed as self-attribution and anticipated attribution by others) on the one hand and prevalence of anxiety, depression, and post-traumatic stress disorder (PTSD) as mental health outcomes on the other hand was analyzed in a population-based survey in Germany.

## Methods

### Data collection and study sample

The present study is based on data from a multi-thematic survey (e.g. psychooncology, quality of life research, sleeping disorder etc.) of the general German population involving several research institutions. As described previously (e.g. [12, 24]), between September and November 2016, a sample of the German general population was examined with the assistance of a demographic consulting company (USUMA, Germany). The entire country was separated into 258 sample areas. Once a sample area was selected, streets, houses, households, and household members were chosen randomly. A first attempt to contact study candidates was made at 4902 addresses, of which 4838 were valid. The subjects were visited by a study assistant. A total of 2510 people between 14 and 93 years old agreed to participate and completed several self-rating questionnaires (participation rate: 51.9% of valid addresses). The reasons for non-participation (48.1%) were: general information request was refused (15.3%), the interview was refused by the target person (14.7%), and there was no one at home for four times in a row (17.2%), as well as other reasons, e. g. illness, vacation (3.2%). All adult participants provided their written informed consent to participate in this study and for the data to be published. Also, written informed consent from the next of kin, caretakers, or guardians on behalf of the minors/children enrolled in the study was obtained. The survey was conducted in German. Subjects with missing information on ‘migration background’ as well as participants with invalid data on self-attribution and/or anticipated attribution by others as an immigrant were excluded from the analysis (*n* = 193). Thus, the final sample consisted of 2317 subjects, with 2069 native Germans (89.3%) and 248 immigrants (10.7%) of 1st (*n* = 115; 46.4% of immigrants sample) and 2nd generation (*n* = 133; 53.6% of immigrants sample). The study was approved by the Ethics Committee of the Medical Faculty of the University of Leipzig.

### Instruments

Sociodemographic data were collected according to the sociodemographic standards of the Federal Statistical Office. In the next step, ‘migration background’ was assessed as defined by official statistics [25]. Participants were asked to provide information about their nationality and their own as well as their parents’ country of birth. In addition, immigration-specific data such as year of immigration and subjective assessments on self and anticipated attributions by others with regard to one’s own ‘migration background’ was recorded (self-attribution – *“Would you describe yourself as an immigrant or person with a migration background?”* and anticipated attribution by others – *“Do you think that others here in Germany would describe you as an immigrant or person with a migration background?”*).

Depression and anxiety were assessed with PHQ-4, a short version of the PHQ-D [26], which consists of two items for generalized anxiety disorder as well as two items for depression according to DSM-IV criteria. Each item can be rated on a 4-point Likert-scale from “not at all” (0) to “nearly every day” (3). Two sum-scores are estimated, one for anxiety (0–6) and one for depression (0–6), adding up the respective items.

Post-traumatic stress disorder was assessed with PCL-5 (PTSD Checklist) [27], a 20-item self-report instrument assessing the symptoms of PTSD as defined in the DSM-5. It was administered together with the revised Life Events Checklist for DSM-5 (LEC-5) for assessing trauma exposure. The 20 items of the PCL-5 can be rated on a 5-point Likert-scale ranging from “not at all” (0) to “extremely” (4), referring to symptoms during the last month. A total score (0–80) can be obtained by summing the scores for each of the 20 items. A provisional PTSD diagnosis may be obtained by taking items rated 2 = “Moderately” or higher into consideration following the PTSD diagnostically rule of the DSM-5: one B item (items 1–5), one C item (items 6–7), two D items (items 8–14) and two E items (items 15–20). In the present study PTSD was assessed by following the diagnostic algorithm according to the DSM-5.

### Statistical analyses

The statistical data analysis was performed with SPSS for Windows version 24. To compare the samples of immigrants and German natives, descriptive statistics and chi-square tests were used. Binary logistic regression models were applied for analyzing possible prediction of immigration-related characteristics on anxiety, depression, and PTSD.

## Results

### Sociodemographic and immigration-related characteristics of the sample

Table 1 gives an overview of the socio-demographic characteristics of immigrants (according to the official definition of persons with ‘migration background’) and native-born Germans. Persons with ‘migration background’ are younger, less often married, more often unemployed and are less likely to live in rural areas, which is in line with Federal statistic reports on immigrant populations in Germany [21].

**Table 1:** Sociodemographic characteristics of the total sample

	German natives *N* = 2069 (89.3%)	Immigrants *N* = 248 (10.7%)	χ^2^
Age			64.36***
*M / SD*	50.6 / 17.6	41.2 / 14.6	
14–34 years	467 (22.6%)	92 (37.1%)	
35–60 years	943 (45.6%)	135 (54.4%)	
ab 61 years	659 (31.9%)	21 (8.5%)	
Sex			0.39
Male	958 (46.3%)	120 (48.4%)	
Female	1111 (53.7%)	128 (51.6%)	
Marital status			23.97***
Married	988 (47.7%)	104 (41.9%)	
Single	581 (28.1%)	92 (37.1%)	
Divorced	286 (13.8%)	45 (18.1%)	
Widowed	207 (10.0%)	7 (2.8%)	
Missing	7 (0.3%)	–	
Partnership			1.23
Yes	1174 (56.7%)	131 (52.8%)	
No	879 (42.5%)	114 (46.0%)	
Missing	16 (0.8%)	3 (1.2%)	
Education			3.57^1^
A-level	450 (21.7%)	67 (27.0%)	
Non A-level	1612 (77.9%)	180 (72.6%)	
Missing	7 (0.3%)	1 (0.4%)	
Employment status			24.25***
Employed	1958 (94.6%)	217 (87.5%)	
Unemployed	95 (4.6%)	30 (12.1%)	
Missing	16 (0.8%)	1 (0.4%)	
Household income			2.39
< 750 Euro	64 (3.1%)	10 (4.0%)	
750–1250 Euro	272 (13.1%)	31 (12.5%)	
1250–2000 Euro	451 (21.8%)	61 (24.6%)	
> 2000 Euro	1206 (58.3%)	132 (53.2%)	
Missing	76 (3.7%)	14 (5.6%)	
Area of residence			9.04**
Rural area	268 (13.0%)	18 (7.3%)	
Small city	200 (9.7%)	18 (7.3%)	
Metropolis	1.601 (77.4%)	212 (85.5%)	
PTSD			0.23
Yes	102 (4.9%)	14 (5.6%)	
No	1945 (94.0%)	232 (93.5%)	
Missing	22 (1.1%)	2 (0.8%)	
Anxiety			2.70
Yes	148 (7.2%)	25 (10.1%)	
No	1915 (92.6%)	223 (89.9%)	
Missing	6 (0.3%)	–	
Depression			0.35
Yes	146 (7.1%)	20 (8.1%)	
No	1911 (92.4%)	226 (91.1%)	
Missing	12 (0.6%)	2 (0.8%)	

***p < .001; **p < .01; ^1^*p* = .059

Table 2 provides detailed characteristics of persons with ‘migration background’ stratified by 1st and 2nd generation immigrants. The vast majority of participants with ‘migration background’ (71.8%) are German citizens, with 53.6% being born in Germany and consequently classified as 2nd generation immigrants. Because of the heterogeneity of the immigrant sample regarding country of origin, which leads to small subgroup sizes, we merged participants to groups of geographic regions, with the exception of participants from Turkey and Poland. About a quarter of persons with ‘migration background’ indicated South-Western EU countries as their region of origin, 16.1% came from Turkey, and 12.1% were from Poland. The mean age at immigration of 1st generation immigrants was reported to be 22 years and they had lived in Germany for 25 years. Nearly two thirds of all persons with ‘migration background’ report both parents having immigrated to Germany.

**Table 2:** Immigration-related characteristics of the 1st and 2nd generation immigrants

	Immigrants total *N* = 248 (100%)	1st Generation *N* = 115 (46.4%)	2nd Generation *N* = 133 (53.6%)	χ^2^
Age at immigration				
M / SD	–	22.45 / 13.07	–	
Length of stay				
M / SD	–	25.01 / 14.65	–	
Parents immigration status				78.60**
One immigrant parent	84 (33.9%)	6 (5.2%)	78 (58.6%)	
Both immigrant parents	164 (66.1%)	109 (94.8%)	55 (41.4%)	
German citizenship				56.38**
Yes	178 (71.8%)	56 (48.7%)	122 (91.7%)	
No	70 (28.2%)	59 (51.3%)	11 (8.3%)	
Country/region of origin				18.19*
Poland	30 (12.1%)	13 (11.3%)	17 (12.8%)	
Turkey	40 (16.1%)	19 (16.5%)	21 (15.8%)	
Former Yugoslavia^a^	13 (5.2%)	6 (5.2%)	7 (5.3%)	
Former Soviet Union^b^	31 (12.5%)	23 (20.0%)	8 (6.0%)	
South-western EU countries^c^	60 (24.2%)	22 (19.1%)	38 (28.6%)	
Eastern EU countries^d^	23 (9.3%)	10 (8.7%)	13 (9.8%)	
African countries^e^	12 (4.8%)	6 (5.2%)	6 (4.5%)	
Middle East countries^f^	15 (6.0%)	6 (5.2%)	9 (6.8%)	
Far Eastern countries^g^	10 (4.0%)	7 (6.1%)	3 (2.3%)	
Other^h^	14 (5.6%)	3 (2.6%)	11 (8.3%)	
Self-attribution as immigrant				51.19**
Yes	85 (34.3%)	66 (57.4%)	19 (14.3%)	
No	161 (64.9%)	48 (41.7%)	113 (85.0%)	
Missing	2 (0.8%)	1 (0.9%)	1 (0.8%)	
Anticipated attribution by others as immigrant				46.48**
Yes	109 (44.0%)	77 (67.0%)	32 (24.1%)	
No	137 (55.2%)	37 (32.2%)	100 (75.2%)	
Missing	2 (0.8%)	1 (0.9%)	1 (0.8%)	

**p < .001; *p < .05; ^a^Albania, Bosnia-Herzegovina, Kosovo, Croatia, Macedonia, Serbia; ^b^Kazakhstan, Kirgizstan, Latvia, Lithuania, Russian Federation, Ukraine, Uzbekistan; ^c^Belgium, France, Greece, Great Britain, Ireland, Italy, Luxemburg, Netherlands, Austria, Switzerland, Spain, Sweden; ^d^Bulgaria, Romania, Slovenia, Czech Republic, Hungary; ^e^Egypt, Algeria, Ghana, Kenia, Democratic Republic of Congo, Morocco, Senegal, Tunis; ^f^Afghanistan, Iraq, Iran, Jordan, Lebanon, Pakistan, Syria; ^g^Bangladesh, China, India, Philippines, Sri Lanka, Thailand, Vietnam; ^h^Argentinia, Australia, Brazil, Chile, USA

About two thirds (64.9%) in total, 41.7% of the 1st generation and 85.0% of the 2nd generation immigrants, denied the attribution of being described as an immigrant from their subjective perspective; 55.2% in total, 32.2% of the 1st generation and 75.2% of the 2nd generation immigrants did not anticipate the attribution of one’s own ‘migration background’ by others. There are significant differences between 1st and 2nd generation immigrants concerning parents’ immigration status (94.8% of 1st generation vs. 41.4% of 2nd generation immigrants with both immigrant parents, χ^2^(1, *N* = 248) = 78.60, *p* < .001), having German citizenship (48.7% of 1st generation vs. 91.7% of 2nd generation immigrants, χ^2^ (1, *N* = 248) = 56.38, *p* < .001), country/region of origin (e.g. 20% of 1st generation vs. 6% of 2nd generation immigrants from the Former Soviet Union, χ^2^(9, *N* = 248) = 18.19, *p* < .05), self-attribution as an immigrant (57.4% of 1st generation vs. 14.3% of 2nd generation immigrants, χ^2^(1, *N* = 246) = 51.19, *p* < .001) and anticipated attribution by others as an immigrant (67% of 1st generation vs. 24.1% of 2nd generation immigrants, χ^2^(1, *N* = 248) = 46.48, *p* < .001).

**Fig. 1:**
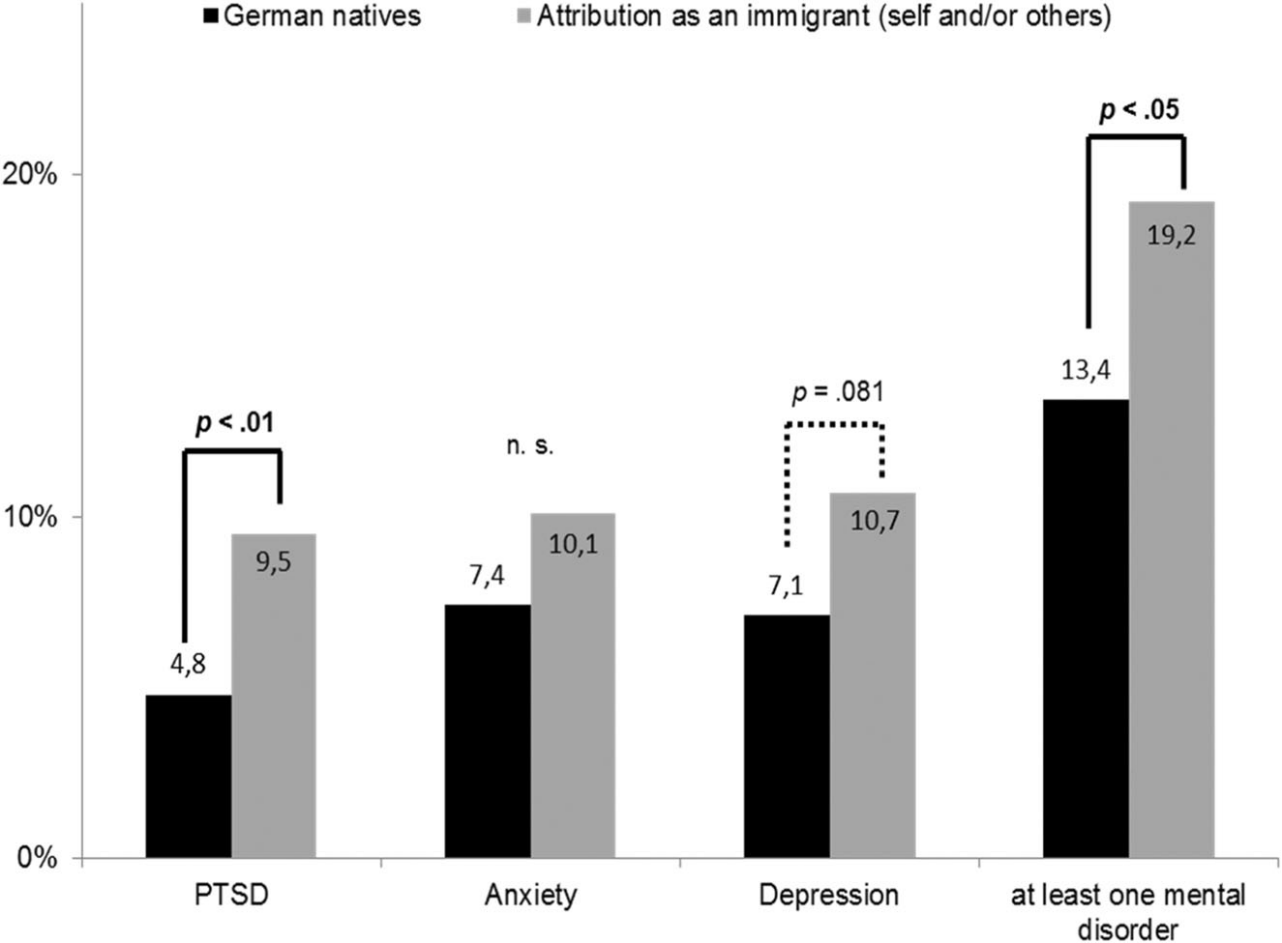
Prevalence rates for post-traumatic stress disorder (PTSD), anxiety, depression and at least one mental disorder among German natives and participants with attribution as an immigrant (self and/or others)

No significant differences between natives and participants with ‘migration background’ were found concerning prevalence rates for anxiety, depression and PTSD (see Table 1). In the next step, we compared prevalence rates for anxiety, depression, PTSD and at least one of these mental disorders of German natives and participants with an attribution of being an immigrant (self-attribution and/or by others). There were significant differences for PTSD (χ^2^(1, *N* = 2214) = 6.98, *p* < .01) and at least one of these mental disorders χ^2^(1, *N* = 2205) = 4.31, *p* < .05), both with lower rates for German natives. The results are shown in Fig. 1.

### Prediction of anxiety, depression and PTSD

To analyse the possible impact of immigration-related factors on mental health status in a ‘migration background’ sample, three binary logistic regression models, each for anxiety, depression and PTSD, were run. The following predictors were put in each model: age, sex, immigrant generation (1st vs. 2nd generation), German citizenship, parents’ immigration status (one immigrant parent vs. two immigrant parents), and attribution as immigrant (self and/or by others vs. neither self-attribution nor attribution by others). The results are displayed in Table 3.

**Table 3:** Post-traumatic stress disorder, anxiety and depression predicted by immigration-related characteristics

Predictor	PTSD		Anxiety		Depression	
	OR / (95% CI)	*p*	OR / (95% CI)	*p*	OR / (95% CI)	*p*
Age	1.03 / (0.97–1.06)	.578	1.03 / (0.99–1.06)	.119	1.01 / (0.97–1.05)	.699
Sex^a,f^	0.20 / (0.05–0.77)	.019	0.97 / (0.42–2.28)	.954	0.96 / (0.37–2.45)	.911
Immigrant generation^b,f^	1.26 / (0.25–6.30)	.781	3.26 / (0.87–12.17)	.078	4.32 / (0.90–20.70)	.067
German citizenship^c,f^	0.22 / (0.04–1.24)	.087	1.25 / (0.38–4.09)	.709	2.08 / (0.54–8.03)	.288
Parents immigration status^d,f^	0.72 / (0.17–3.05)	.659	0.83 / (0.30–2.30)	.717	0.45 / (0.14–1.46)	.183
Attribution as immigrant (self and/or by others)^e,f^	4.05 / (1.06–15.46)	.040	1.47 / (0.54–3.98)	.452	3.88 / (1.29–11.89)	.017

^a^male = 1, female = 2; ^b^1st generation = 1, 2nd generation = 2; ^c^yes = 1, no = 2; ^d^one immigrant parent = 1, both immigrant parents = 2; ^e^no = 0, yes = 1; ^f^the lower category represents the reference category

Predicting PTSD among ‘persons with migration background’, significant influences were found for sex and attribution as an immigrant. According to this, male ‘persons with migration background’ (OR: 0.20, 95% CI: 0.05–0.77) and those who consider themselves as an immigrant and/or anticipate the attribution as an immigrant by others (OR: 4.05, 95% CI: 1.06–15.46) are more likely to suffer from PTSD. In addition, self-attribution and/or attribution by others as an immigrant was a significant predictor for depression, with higher rates in participants with ‘migration background’ who see themselves as an immigrant or anticipate being described as an immigrant by others (OR: 3.88, 95% CI: 1.29–11.89). No significant effects were found for anxiety.

## Discussion

In the present study, anxiety, depression, and PTSD were assessed in natives and immigrant populations in Germany using a population-based approach. Although there are some significant differences between 1st and 2nd generation immigrants regarding immigration-related characteristics, no differences in prevalence rates for PTSD, anxiety and depression were found between persons with ‘migration background’ and German natives. In contrast, prevalence rates for PTSD and at least one mental disorder were found to be higher in participants who consider themselves as an immigrant and/or anticipate the attribution by others compared to German natives. At this point, the findings are in line with previous research showing no differences between self-reported mental health among persons with ‘migration background’ and natives in Germany [1, 3, 7, 10]. However, several studies reported better health [28–31] in immigrants; some other studies show worse mental health in immigrant populations [6, 8, 9] compared to native-born participants. Some studies underpinned the role of immigration-related factors, especially of young age at immigration [10, 28, 31], country of origin [1, 28, 31–34], length of stay in the host country, and consequently the immigrant generation [10, 35]. Additionally, in the present study the prevalence rates for PTSD, anxiety, and depression were analysed by considering self-attribution as well as attribution by others on one’s own ‘migration background’, which – in contrast to the official, criteria-based definition of a person with a ‘migration background’ – seem to better differentiate between groups of immigrants suffering from mental health problems. Self-attribution and/or anticipated attribution by others on one’s own ‘migration background’ were found to be associated with higher rates for PTSD and depression. To our knowledge, the present study is the first one focusing on mental health outcomes by paying attention to the subjective perspective of the participants’ immigration status. This novelty unfortunately makes a comparison of our results with previous research impossible. Consequently, studies in different host countries should be conducted with special emphasis on self-attribution as well as anticipated attribution by others on one’s own immigration status. Since individuals with a ‘migration background’ represent an extremely heterogeneous group in Germany, rather small subgroups of immigrants from different origins can be found in population surveys. This severely limits the statistically verified statements about the differences between people from different origins. Thus, in the context of this work, it was not possible to carry out country-of-origin-specific analyses in terms of possible cultural and/or ethnic characteristics. Particularly with regard to the question addressing self- and/or external attribution on one’s own ‘migration background’, the information on cultural distance to the host country (e.g. Polish vs. Vietnamese vs. Turkish immigrants in Germany) could have an influence on mental health outcomes. Future work should therefore aim at larger samples of persons with ‘migration background’ than it was possible in the present study. In addition, further, more in-depth analyses should cover perceived discrimination among participants with ‘migration background’ as well as cultural and/or ethnic identity. It should be noted that the questions asked about self- and/or anticipated attribution by others on one’s own ‘migration background’ might be answered in light of (1) a possibly negatively attributed connotation of the terms ‘immigrant’ and/or ‘migration background’, and/or (2) in light of strong identification with a particular cultural or ethnic group. This aspect should therefore be taken into account when interpreting the results of the present work. Moreover, in the present study we decided to analyse the impact of subjective attribution on mental health in general, mostly because of the rather explorative nature of the collected data. For the further research larger data sets are needed to better differentiate between self and external attribution on one’s own migrations background within the subgroup of immigrants suffering from mental health problems.

Although the study has some major strengths (e.g. population-based approach, large sample size, inclusion of immigration-related factors), there are some factors, which limit how our results can be interpreted and consequently illuminate some other important implications for future research. The data is supposed to be representative, but the proportion of immigrants in our dataset is in fact lower than found in the general population of Germany – 10.7%. vs. 22.5% [25], which could be explained by the fact that the minimum age of participation at this study was 14 years, and the proportion of children with a ‘migration background’ is generally higher, e.g. 36% of children under the age of 5 years have a ‘migration background’ according to the official statistics [36]. Looking at socio-demographic and immigration-related characteristics, the subgroup of persons with ‘migration background’ in the present study is similar to the general population. However, our study includes mostly well-integrated immigrants, who have been living in Germany for years or even since their birth. Due to the methodology used (e.g. interviews were performed in German only), it was hardly possible to include immigrants with poor language skills, refugees, illegal immigrants as well as illiterate persons. On the other hand, to the best of our knowledge, our study represents the first population-based analysis on subjective perspective of the participants’ immigration status having impact on mental health based on a large and heterogeneous subgroup of immigrants.

Overall, it can be concluded that the results of this work suggest a more specific examination of the usual operationalization of the term ‘migration background’. It seems particularly important to consider self-attribution and/or anticipated attribution by others on one’s own ‘migration background’ by answering research questions which are linked to subjective perspective of the individual, as it happens in the fields of personality assessments or self-reported mental health issues. Especially in these areas of research there are often inconsistent findings, e.g. partially contradictory reports on health-related advantages or disadvantages among immigrants compared to natives in different host countries [1, 7, 12]. It is therefore important to differentiate between the official definition and the subjective attribution of membership to one or another population group. Finally, because of lacking research until now, the impact of self-attribution and/or anticipated attribution by others with respect to one’s own ‘migration background’ on mental health outcomes cannot yet be clearly answered, although the results of the present study provide first important approaches for in-depth, further analyses. Moreover, there is need for empirical studies explaining the relationship between subjective and objective perceptions on immigration status and mental health in immigrant populations to build up a robust theoretical frame for possible mechanisms on it.

## Data Availability

The datasets generated and/or analysed during the current study are not publicly available due to ongoing analyses, but are available from the corresponding author on reasonable request.
